# 377. Closing the Gap in Clostridioides difficile Infection Prevention: An EHR Driven Approach to Oral Vancomycin Optimization

**DOI:** 10.1093/ofid/ofaf695.124

**Published:** 2026-01-11

**Authors:** Naida Koura-Mola, Hongkai Bao, Mei H Chang, Priya Nori, Kelsie Cowman, Yi Guo, Terrence McSweeney

**Affiliations:** Montefiore Einstein, Bronx, New York; Montefiore Medical Center, Bronx, New York; Montefiore Einstein, Bronx, New York; Montefiore Health System, Bronx, NY; Montefiore Medical Center, Bronx, New York; Montefiore Medical Center, Bronx, New York; Montefiore Einstein, Bronx, New York

## Abstract

**Background:**

Systemic antibiotics significantly increase the risk of Clostridioides difficile infection (CDI) recurrence, particularly within the first 2-4 months following an initial episode. Studies have demonstrated that low-dose oral vancomycin prophylaxis (OVP) reduces CDI recurrence in these patients. Although OVP is recommended in our institution protocol, adherence has been inconsistent. To improve OVP prescribing, we implemented an electronic health record (EHR) embedded CDI prophylaxis order set.

**Figure:**
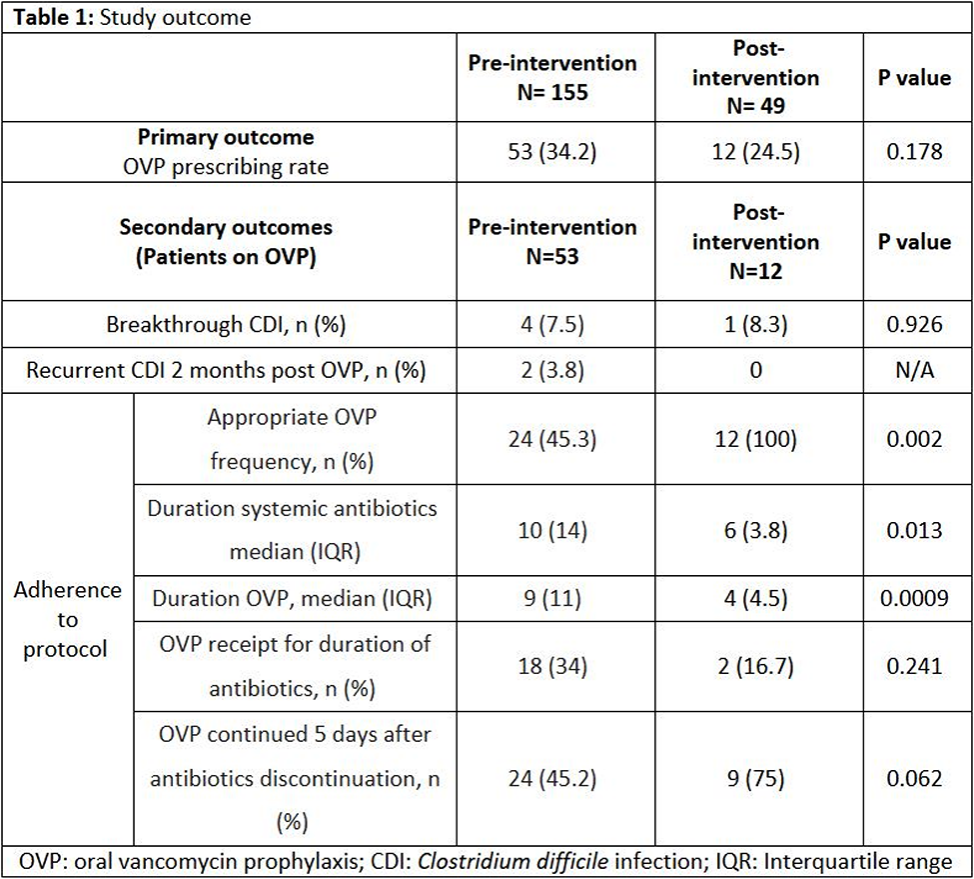

**Figure:**
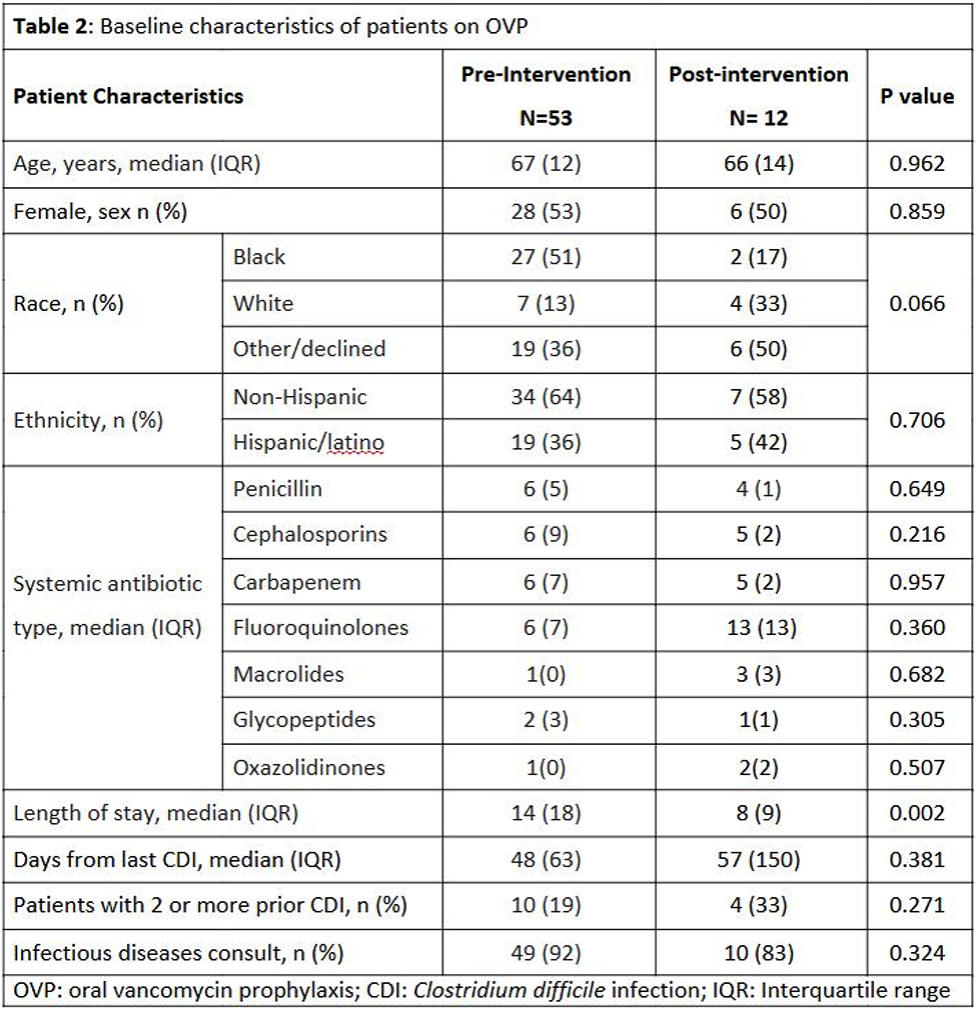

**Methods:**

This single-center, quasi-experimental, performance improvement project evaluated adults prescribed systemic antibiotics with a positive Clostridioides difficile PCR or toxin test within the past 12 months. The primary outcome was OVP prescribing rate pre- and post- order set implementation. Secondary outcomes included adherence to institutional protocol, rates of CDI breakthrough or recurrence, systemic antibiotic duration, and length of stay (LOS).

**Results:**

A total of 204 patients were included, with 155 in the pre-intervention group and 49 in the post-order set group, (table 1). OVP prescribing rates were similar between groups (34.2% vs 24.5%, P= 0.178). Among patients who received OVP with systemic antibiotics, adherence to institutional protocol for OVP frequency significantly improved in the post- order set group compared to the pre-intervention group (100% vs 45.3%, P=0.002). Breakthrough CDI occurred in 4 patients pre-intervention and 1 patient post-intervention, while recurrent CDI within 2 months occurred in 2 and 0 patients, respectively. Median LOS decreased from 14 to 8 days (P= 0.002), and median systemic antibiotic duration decreased from 10 to 6 days (P= 0.013) in the pre-intervention group and the post-order set group, respectively, (table 2).

**Conclusion:**

Implementation of an EHR embedded CDI prophylaxis order set did not increase OVP prescribing rates but successfully standardized prescribing practices. Introducing an EHR best-practice advisory (BPA) is the next step to further enhance OVP utilization in patients with a history of CDI receiving systemic antibiotics.

**Disclosures:**

All Authors: No reported disclosures

